# Smartphone-based vestibular & gait analysis for remote fall risk assessment

**DOI:** 10.3389/fdgth.2026.1798559

**Published:** 2026-07-02

**Authors:** Eugene Rezk, Marion Luschin-Ebengreuth, Hannes Kaufmann

**Affiliations:** 1 Virtual and Augmented Reality Group, Institute for Visual Computing and Human-Centered Technology, Faculty of Informatics, TU Vienna, Vienna, Austria; 2 Department of Orthopedics and Traumatology, Military Hospital Vienna, Vienna, Austria; 3 Department of Medical Physics and Biophysics, Medical University of Graz, Graz, Austria; 4 Entrepreneurship in Digital Health [EDITH] (CE), U089 Medical University of Graz, Graz, Austria; 5 Department of Plastic Surgery, Medical University of Graz, Graz, Austria

**Keywords:** computational modeling, digital biomarkers, gait analysis, smartphone-based sensing, inertial measurement unit (IMU), multimodal signal processing, simulation-based framework, vestibular assessment

## Abstract

Dizziness and vertigo are among the most common reasons for clinical consultation worldwide and represent key risk factors for falls, particularly in neurologic and neuroorthopedic patient populations. Early identification of balance and gait impairments is therefore relevant for fall prevention. However, access to established diagnostic tools such as video-oculography (VOG) and motion analysis systems remains limited due to cost, technical complexity, and the need for specialized personnel, especially in resource-constrained settings. This work proposes a conceptual framework for smartphone-based assessment of vestibular function and gait stability, aimed at supporting fall-related screening concepts. The approach is designed to integrate near-infrared eye tracking with inertial measurement unit (IMU)-based motion analysis within a unified smartphone-based sensing environment, enabling mobile and scalable applications. The framework outlines methods for extracting oculomotor and biomechanical features, including vestibulo-ocular behavior and gait stability metrics such as Triangular Area Variability (TAV) and inter-limb symmetry. In addition, it introduces the use of virtual anatomical reference points (S1, $P_{L}$, $P_{R}$) and the projection of sacral motion onto the interpatellar axis to describe biomechanical stability. As a conceptual contribution, this work does not include clinical validation or real-world data but provides a structured methodological foundation for future studies. The proposed framework is intended to support the development of accessible, smartphone-based screening and monitoring approaches for the early identification of balance-related impairments, thereby contributing to fall prevention research in neurologic and neuroorthopedic populations.

## 1 Related work

Dizziness and vertigo represent a major clinical burden worldwide, with Benign Paroxysmal Positional Vertigo (BPPV) being among the most prevalent vestibular disorders [1, 2]. Established diagnostic procedures rely on clinician-administered maneuvers such as the Dix–Hallpike test, combined with visual assessment of nystagmus. While effective, these approaches are inherently subjective and require trained specialists. Video-oculography (VOG) has emerged as a gold-standard technique for objective measurement of eye movements, enabling precise quantification of nystagmus characteristics. However, VOG systems are associated with substantial financial cost, specialized hardware requirements, and limited availability outside tertiary care centers [3]. This creates a significant accessibility gap, particularly in rural and resource-constrained settings. Recent advances in mobile health technologies have explored the use of smartphone-based sensors for neurological and vestibular assessment [4, 5]. Approaches combining inertial measurement units (IMUs), camera-based tracking, and machine learning have shown potential for remote monitoring and telemedical applications [6, 7]. In parallel, eye-tracking methods based on infrared imaging and computer vision algorithms have been proposed to approximate laboratory-grade measurements using consumer hardware [8]. In the domain of gait and balance assessment, clinical tools such as the Tinetti Performance-Oriented Mobility Assessment remain widely used for fall risk evaluation [9]. More recently, research has focused on translating such assessments into sensor-based metrics derived from wearable or mobile devices, enabling quantitative and continuous monitoring. Despite these developments, a unified framework that integrates eye movement analysis and gait-based stability metrics into a single, accessible smartphone-based system remains insufficiently explored. The present work addresses this gap by proposing a simulation-based conceptual proof-of-concept architecture combining both modalities.

### 1.1 Clinical reference: Tinetti balance assessment

Clinical fall risk in older adults is commonly assessed using the *Tinetti Performance Oriented Mobility Assessment* (POMA), a widely used clinical instrument for evaluating balance and gait impairments associated with increased fall risk [9]. The balance component consists of standardized tasks performed during sitting, standing, and transitional movements, each scored on an ordinal scale from 0 (severe impairment) to 2 (independent performance), with a maximum score of 16 points. The assessment includes tasks such as sitting balance, rising from a chair, immediate standing stability, response to perturbation, turning, and controlled sitting. These tasks reflect fundamental motor control and postural stability mechanisms relevant to vestibular function. From a systems perspective, the Tinetti assessment provides a structured clinical reference for describing mobility-related performance patterns. The discrete motor tasks can be conceptually related to measurable kinematic features derived from IMU signals, such as symmetry-, variability-, and stability-related descriptors. In the proposed system, geometric descriptors such as Triangular Area Variability (TAV) and inter-limb symmetry are inspired by structural elements of Tinetti balance tasks. This enables a computational representation of selected mobility-related characteristics within a sensor-based and simulation-driven framework, without attempting to replicate the clinical scoring procedure.

## 2 System design

The proposed system is conceptualized as an integrated, multimodal sensing and analysis framework for vestibular and balance assessment, intended for implementation on commodity smartphone hardware. The architecture follows a modular design paradigm, combining vision-based oculomotor analysis with inertial sensor-based biomechanical modeling to enable a structured representation of vestibular-related signals. At the hardware level, the system assumes a smartphone equipped with a near-infrared (NIR) imaging unit. This may either be natively integrated (e.g., structured-light or time-of-flight sensors) or represented via an external clip-on module in a conceptual setup. Operating in the infrared spectrum improves robustness against ambient illumination variability and enhances pupil contrast [10]. To ensure geometric consistency within the conceptual model, a head-mounted stabilization mechanism is assumed to enforce a fixed relative transformation between the camera coordinate system and the ocular reference frame. This relationship can be described by a rigid body transformation (Equation 1)

$$x_{eye}=Rx_{cam}+t,$$ (1)

where R∈SO(3) and $t\in R^{3}$ denote rotation and translation, respectively. This constraint is used to illustrate reduction of motion-induced artifacts and to support consistent geometric assumptions within the simulation framework.

**Figure 1:**
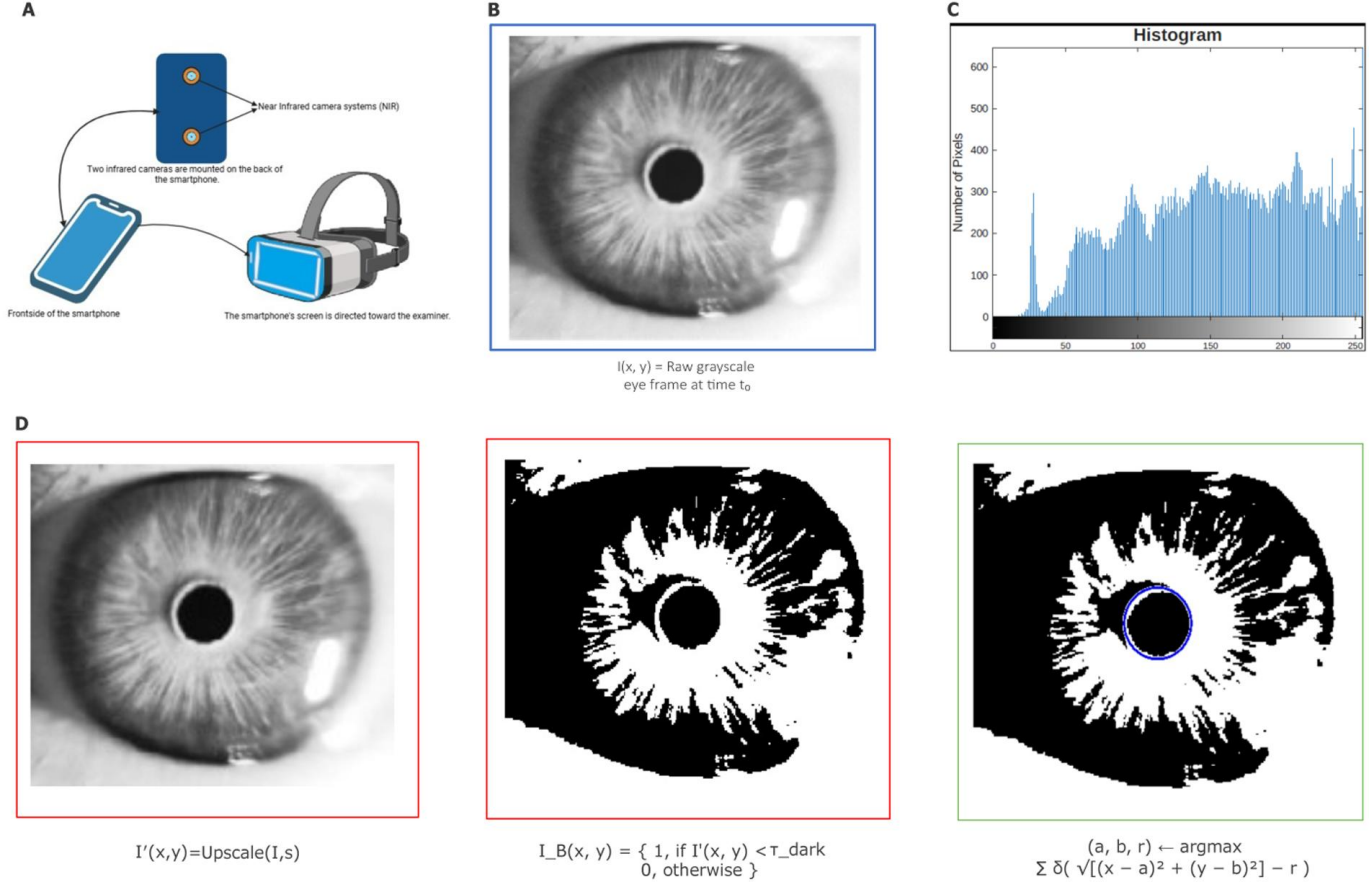
Overview of the proposed oculomotor and gait analysis system. **(A)** Schematic representation of a head-mounted smartphone configuration for eye-tracking. Created using BioRender.com. A near-infrared (NIR) camera system is positioned on the posterior side of the device and aligned toward the subject’s eyes via a stabilized mounting interface. This configuration assumes controlled illumination conditions and a fixed geometric relationship between the sensor and ocular structures, while allowing the examiner to monitor acquisition through the front display. **(B)** Representative raw grayscale infrared image of the eye. Image taken with permission from Haslwanter T. [11]. The pupil appears as a low-intensity region with high contrast relative to the surrounding iris tissue, enabling conceptual segmentation under NIR illumination. **(C)** Intensity histogram of the corresponding infrared image, illustrating the bimodal distribution of pixel intensities. The separation between dark (pupil) and brighter regions forms the basis for thresholding approaches, such as fixed-point iteration, used for illustrative segmentation under varying imaging conditions. **(D)** Preprocessing and segmentation of grayscale infrared eye images, including intensity normalization, adaptive thresholding, and geometric pupil localization, enabling extraction of oculomotor features under simulated imaging conditions.

**Figure 2:**
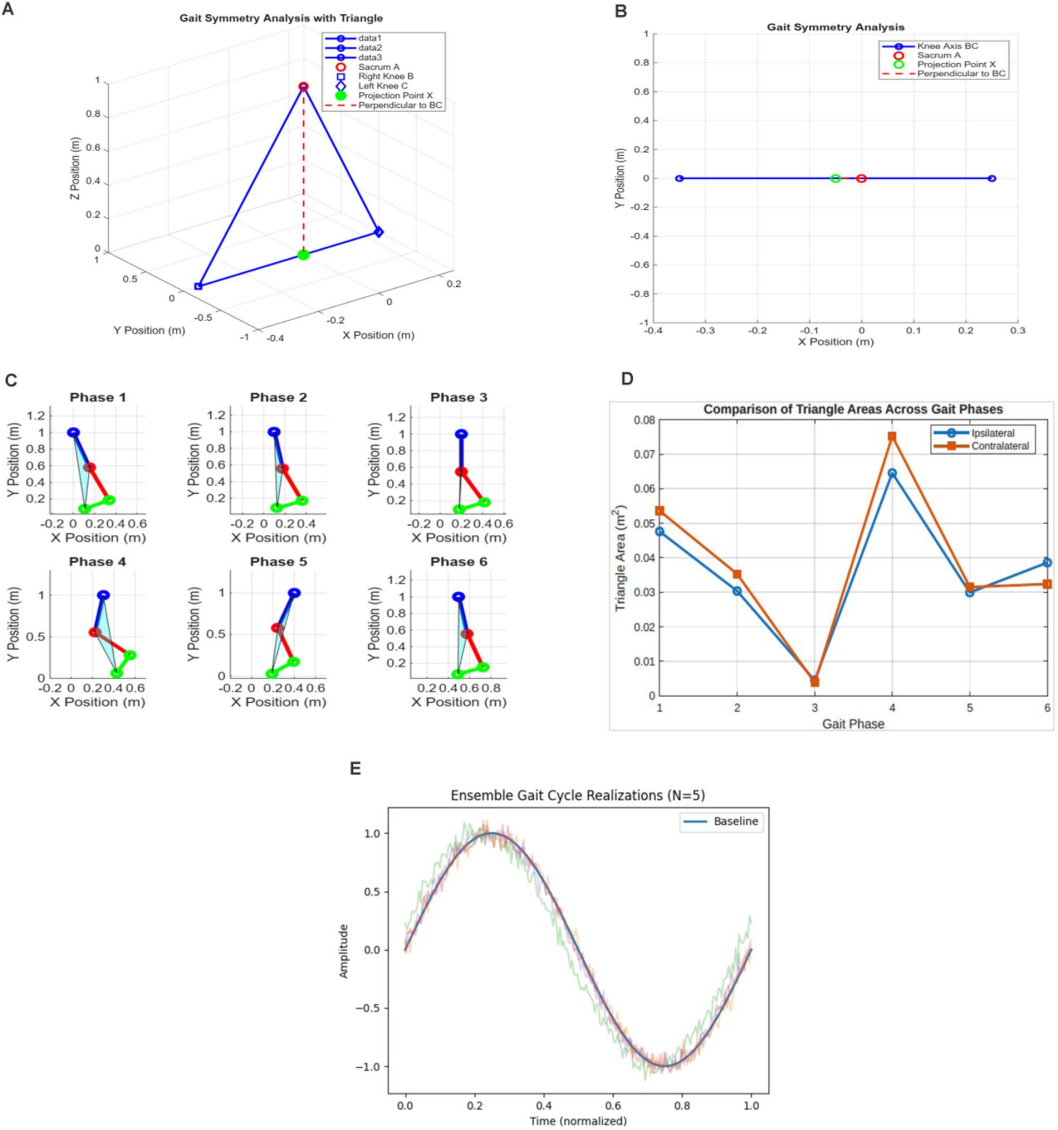
Multimodal pipeline for image-based oculomotor feature extraction and gait-related geometric analysis. **(A)** Geometric representation of a static stance configuration inspired by clinical balance assessment as defined in the Tinetti Performance-Oriented Mobility Assessment (POMA). A triangular configuration based on virtual anatomical landmarks (sacrum and bilateral lower-limb reference points) is used as a conceptual approximation of postural symmetry and stability during standing tasks. **(B)** Two-dimensional projection of the same configuration, highlighting inter-limb symmetry, projection points, and perpendicular distances as simplified descriptors for balance assessment, consistent with clinical scoring principles. **(C)** Phase-resolved visualization of reconstructed gait configurations across discrete gait phases, reflecting functional gait analysis within a conceptual Tinetti-inspired framework. The selected anatomical reference points (sacrum, knee joints, and forefoot positions) are conceptually informed by biomechanical models such as the Virtual Pivot Point (VPP), where the sacral region serves as an approximation of the center of mass (CoM) under constrained sensing conditions. **(D)** Quantitative comparison of triangular area measures for ipsilateral and contralateral configurations across gait phases, illustrating temporal variability and inter-limb asymmetries as descriptive indicators of gait stability. The figure illustrates the integration of clinically inspired balance assessment concepts and simplified biomechanical modeling for the extraction of geometric stability features from multimodal sensor data under simulation-based conditions. **(E)** A baseline periodic signal (bold line) is shown together with a set of phase-shifted and noise-perturbed realizations (N=5, including baseline + 4 perturbations). The perturbed signals are generated from a single gait template and are not independent physiological gait cycles, but stochastic realizations used to emulate measurement variability and temporal perturbations. This ensemble is used to assess the robustness and numerical stability of feature extraction under controlled simulation conditions.

On-device computation is assumed to be supported by heterogeneous processing units, including graphics processing units (GPUs) and neural processing units (NPUs), enabling low-latency inference within a conceptual mobile processing pipeline. A schematic overview of the hardware configuration is provided in Figure 1A, illustrating the smartphone-based acquisition setup with an integrated near-infrared (NIR) imaging system mounted on a head-stabilized interface in the proposed design. The integrated framework is designed to enable the simultaneous extraction of oculomotor and kinematic features, supporting a multimodal functional representation of vestibular and balance-related processes within a simulation-based environment. Functionally, the system is decomposed into two primary subsystems:

(1) **Oculomotor Analysis Module:** This module estimates eye movement trajectories $p(t)\in R^{2}$ from infrared image sequences and derives oculomotor features such as slow-phase velocity, fast-phase frequency, and torsional components for the conceptual characterization of nystagmus-like patterns (Figure 1).
(2) **Inertial Gait Analysis Module:** This module processes inertial measurement unit (IMU) signals, including linear acceleration a(t) and angular velocity ω(t), to estimate kinematic characteristics of human gait in a simulated setting. Based on reconstructed motion representations, geometric stability descriptors are derived to quantify balance and inter-limb coordination within the proposed framework (Figure 2). The system is designed for conceptual autonomous operation in non-clinical environments and may support integration into telemedical workflows, enabling asynchronous expert review and remote decision support in future implementations.

## 3 Implementation

### 3.1 Fixed-point iteration and pupil segmentation

To reduce computational complexity and support stable segmentation behavior in the proposed framework, a fixed-point iteration method is used to convert grayscale infrared eye images into binary masks (Figure 1). This method determines an adaptive threshold by iteratively estimating centroids of intensity distributions. The segmented binary image isolates the pupil region, allowing faster feature extraction in the pipeline.

To localize the pupil in infrared eye images, a fixed-point iteration method is applied to estimate an adaptive global threshold for binarization. Starting from an initial threshold $T_{0}$, the mean intensities $\mu_{1}$ and $\mu_{2}$ of pixels below and above the threshold are iteratively computed:

$$T_{n+1}=\frac{\mu_{1}+\mu_{2}}{2}$$

The iteration proceeds until $|T_{n+1}-T_{n}|<\varepsilon$, where ε is a small convergence criterion (e.g., 1 intensity level). This method enables adaptive segmentation of the pupil under varying illumination conditions, as illustrated in Figure 1D.

The resulting binary mask B(x,y) is defined as:

$$B(x,y)=\left\{ \begin{array}{cc} 1\; & \text{if }I(x,y)<T^{*}\;\\ 0\; & \text{otherwise}\; \end{array}\right.$$

where $T^{*}$ is the converged threshold.

### 3.2 Hough transformation for circle detection

After binarization, the Hough Circle Transform is applied within the proposed framework to detect circular structures corresponding to the pupil [12, 13]. This geometric detection complements intensity-based segmentation and supports extraction of pupil-related features. The Hough method provides a parametric representation that is used for circle localization in the image domain. To detect circular shapes corresponding to the pupil in the binary image B(x,y), the Hough Circle Transform is applied. The method operates in a parametric space defined by the circle equation:

$$(x-a{)}^{2}+(y-b{)}^{2}=r^{2}$$

where (a,b) is the center and r the radius of the circle.

Each edge point $\left(x_{i},y_{i}\right)$ in the binary image votes in an accumulator space for possible circle centers (a,b) at a given radius r.

The algorithm proceeds as follows:

1. For each edge point $\left(x_{i},y_{i}\right)$ where $B(x_{i},y_{i})=1$:
2. For each radius r in a predefined range:
3. Update the accumulator array A(a,b,r) where:
  $$a=x_{i}-r\cos\theta ,\;b=y_{i}-r\sin\theta ,\;\theta \in [0,2\pi )$$

The maxima in A(a,b,r) correspond to candidate circle parameters in the image. The tuple $\left(a^{*},b^{*},r^{*}\right)$ with the highest votes defines the detected pupil contour within the proposed framework. This method provides a structured geometric representation of pupil shape under simulated infrared imaging conditions.

### 3.3 IMU-based gait analysis

To assess the conceptual feasibility of gait analysis using smartphone-based inertial measurement unit (IMU) data, a proof-of-concept pipeline is proposed using simulated IMU signals sampled at 50 Hz (Figure 2). The primary objective is to verify the internal consistency and numerical stability of the signal processing pipeline, as well as the stability and reproducibility of the derived biomechanical features. Sensor orientation is estimated using the Madgwick sensor fusion algorithm, yielding roll and pitch angles for gravity compensation within the simulation framework. Following orientation correction, a virtual anatomical reference point approximating the sacrum, denoted as $\vec{S}(t)$, is reconstructed through numerical integration of the projected acceleration signals, combined with filtering to reduce drift effects. Bilateral knee joint trajectories, denoted by ${\vec{P}}_{L}(t)$ and ${\vec{P}}_{R}(t)$, are approximated to construct a simplified representation of gait dynamics. The proposed modeling framework is conceptually related to established clinical and biomechanical gait assessment approaches. In particular, it is inspired by the structure of the Tinetti Performance-Oriented Mobility Assessment, a widely used clinical tool for evaluating gait and balance performance in older adults [9, 14]. The derived features are not intended to replicate clinical scoring, but to represent computational analogues of gait symmetry and stability components that are implicitly reflected in such assessments. From a biomechanical perspective, the framework is informed by the Virtual Pivot Point (VPP) concept, which models human gait as a control strategy directing ground reaction forces toward a virtual point above the center of mass [15–17]. This abstraction captures essential aspects of postural control while remaining computationally efficient within a simplified modeling environment. Compared to more detailed neuromechanical models—such as inverted pendulum formulations, central pattern generator (CPG) approaches, or full musculoskeletal simulations [18]—the proposed representation deliberately prioritizes simplicity and simulation-based feasibility for feature exploration in IMU-derived signals. Alternative gait modeling approaches, including linear inverted pendulum models (LIPM), zero moment point (ZMP)-based formulations, and data-driven machine learning models, typically require stronger physical assumptions or large annotated datasets. In contrast, the present approach emphasizes a compact, feature-driven representation intended for exploratory analysis within a simulated IMU environment, while capturing structural properties of gait behavior inspired by Tinetti-like assessment principles and VPP-based stability concepts. Based on these virtual landmarks, geometric descriptors of gait stability and symmetry are derived. The primary feature, defined as the *Triangular Area Variability* (TAV), is given by Equation 2:

$$A(t)=\frac{1}{2}\left\|\left({\vec{P}}_{L}(t)-\vec{S}(t)\right)\times \left({\vec{P}}_{R}(t)-\vec{S}(t)\right)\right\|$$ (2)

To enable a surrogate representation of stride-to-stride variability, A(t) is evaluated over an ensemble of phase-shifted and noise-perturbed realizations of the base cycle. Additional complementary features are computed to characterize inter-limb symmetry (Equation 3) and angular coordination (Equation 4): ?>

$$\Delta_{\mathrm{sym}}(t)=\left|\left\|\vec{S}(t)-{\vec{P}}_{L}(t)\right\|-\left\|\vec{S}(t)-{\vec{P}}_{R}(t)\right\|\right|$$ (3)

$$\theta (t)=\cos^{-1}\left(\frac{{\vec{v}}_{L}(t)\cdot {\vec{v}}_{R}(t)}{\left\|{\vec{v}}_{L}(t)\right\|\left\|{\vec{v}}_{R}(t)\right\|}\right)$$ (4)

where ${\vec{v}}_{L}(t)$ and ${\vec{v}}_{R}(t)$ denote the instantaneous velocity vectors of the left and right knee trajectories, respectively. The proposed pipeline was evaluated in a simulation-based setting using a synthetic gait cycle. To approximate stride-to-stride variability, the base cycle was treated as a generative template from which a limited set of phase-shifted and noise-perturbed realizations (N=5) was derived, enabling assessment of numerical stability and consistency of feature extraction under controlled conditions.

#### 3.3.1 Geometric feature formulation

The derived features are based on a simplified geometric representation of anatomical landmarks, including the sacrum and bilateral knee positions. These points define a dynamic triangular configuration, from which multiple features are derived within the proposed framework. In addition to the triangular area, symmetry is quantified by comparing distances between the sacrum and bilateral landmarks, while angular relationships between limb vectors provide information on coordination and relative spatial configuration. This geometric formulation is conceptually related to established biomechanical modeling ideas, such as the Virtual Pivot Point (VPP), where the sacral region serves as a simplified reference point under constrained sensing assumptions. The presented features are intentionally designed to balance computational simplicity with geometric interpretability, enabling their use in a simulation-based and resource-aware analysis setting.

### 3.4 Deep learning-based classification

To enable sequence-based analysis of oculomotor patterns, a conceptual learning approach is proposed that targets the temporal structure of nystagmus-like eye movement signals. Rather than relying solely on pre-defined summary statistics, the model is designed to operate on temporally ordered representations derived from infrared image sequences and corresponding pupil trajectories [8]. This design aims to represent both local fluctuations and broader oscillatory patterns within simulated eye movement data. The proposed input representation consists of synchronized frame-wise image data and derived motion descriptors, including pupil displacement, velocity profiles, and slow-phase characteristics [19]. Particular emphasis is placed on slow-phase velocity (SPV) and the alternation between slow and fast phases, as descriptive components of vestibular-related signal patterns within the simulation framework. Spatial feature extraction is intended to operate directly on infrared eye images to preserve information on pupil shape, partial occlusions, and illumination-dependent variations, which may influence signal representation quality in simulated acquisition conditions. Temporal modeling is conceived to focus on the sequential structure of eye movement signals. In contrast to static classifiers, the proposed approach is intended to represent: (i) periodicity of oscillatory patterns, (ii) phase transitions between slow and fast components, and (iii) temporal asymmetries in simulated nystagmus-like signals. In addition to implicit feature learning, frequency-related characteristics are intended to be represented by exposing the model to signals containing dominant oscillatory components, allowing it to learn associations between temporal patterns and signal dynamics within the simulated dataset, without enforcing hard-coded physiological thresholds. The classification output is defined in a conceptual manner to distinguish between different simulated eye movement pattern classes (e.g., regular vs. irregular oscillatory behavior). The architecture is structured as a flexible framework for potential future extension toward finer-grained categorization. Importantly, this work focuses on the conceptual architecture and its integration within a multimodal simulation framework. Model training, dataset curation, and quantitative performance evaluation are not part of this proof-of-concept study and are deferred to future work.

## 4 Evaluation

The proposed system was evaluated in a controlled simulation environment with the primary objective of verifying the computational correctness, numerical stability, and internal consistency of the processing pipeline.In this context, the evaluation represents a technical assessment of the framework rather than a clinical or real-world performance evaluation, as no experimental or patient data were used. A synthetic gait cycle was generated to serve as a controlled input for testing the IMU-based signal processing pipeline. To approximate stride-to-stride variability, a limited ensemble (N=5) of temporally perturbed realizations was derived from the base cycle to assess the robustness of the derived features under minimal stochastic variation. Simulated inertial signals, a(t) and ω(t), were used to reconstruct virtual anatomical trajectories, including the sacral reference $\vec{S}(t)$ and bilateral knee joint trajectories ${\vec{P}}_{L}(t)$ and ${\vec{P}}_{R}(t)$. These reconstructed signals were analyzed with respect to numerical stability, drift behavior, and the internal consistency of the derived biomechanical features under simulation conditions.

The evaluation focused on the following criteria:

- Convergence and stability of numerical integration over time
- Behavior of geometric feature extraction under controlled perturbations
- Sensitivity of the derived Triangular Area Variability (TAV) and symmetry-related metrics to signal noise and variations

In addition, the eye-tracking component was assessed using representative infrared image sequences. The evaluation examined the behavior of preprocessing, segmentation, and circular feature detection under varying noise levels and illumination conditions within the simulation setup. No quantitative performance metrics (e.g., accuracy, sensitivity, or specificity) were computed. The results are intended to describe the internal behavior of the algorithmic components within the simulation-based framework. It is emphasized that this evaluation is intended as a proof-of-concept demonstration of pipeline behavior under simulated conditions. A comprehensive clinical validation, including statistical analysis on real-world datasets, is required to assess applicability and generalizability.

## 5 Results

The simulations illustrate the behavior of the proposed multimodal processing pipeline across both kinematic and oculomotor domains. The IMU-based module produces reconstructed trajectories of virtual anatomical landmarks, enabling the computation of geometric descriptors within the simulation framework. Ensemble realizations are used to evaluate feature stability, with results reported in aggregated form for clarity. In particular, the Triangular Area Variability A(t) exhibits consistent temporal behavior under simulated conditions while remaining responsive to induced perturbations. This suggests its potential role as a geometric descriptor for representing gait-related variability within the proposed model. The eye-tracking module produces pupil-related estimates from infrared image sequences under simulated conditions. The combination of intensity-based segmentation and geometric refinement yields estimates of the pupil center p(t) within the defined processing pipeline. As illustrated in Figure 2A, the pipeline transforms raw grayscale infrared images I(x,y) into enhanced representations $I^{\prime }(x,y)$, followed by adaptive binarization B(x,y) via fixed-point iteration and subsequent geometric detection of pupil parameters (a,b,r) using the Hough Circle Transform. This sequence represents the computational flow of the proposed method under simulation conditions. The geometric gait representation, shown in Figures 2C,D, illustrates simplified configurations based on virtual anatomical landmarks (sacrum S, left and right patellae $P_{L}$, $P_{R}$). These landmarks define a triangular structure used for deriving geometric descriptors within the simulation framework. The two-dimensional projection highlights symmetry-related quantities, projection relationships, and distance-based measures as abstract representations of postural configuration. Across the simulated scenarios, the framework enables the extraction of oculomotor and kinematic features within a unified computational representation. The integration of both modalities demonstrates the internal consistency of the proposed pipeline under simulation-based conditions. Overall, the results describe the behavior of the proposed computational framework and support its formulation as a simulation-based proof-of-concept for multimodal feature extraction.

## 6 Discussion and limitations

While the presented framework demonstrates the “conceptual feasibility” of a multimodal, smartphone-based vestibular assessment system, several important limitations must be critically discussed. First, the current study is entirely simulation-based and does not include experimental validation with human subjects. All biomechanical and inertial signals are synthetically generated, and the eye-tracking pipeline is evaluated only on representative or illustrative data. Consequently, the external validity of the proposed system remains limited, and no conclusions regarding clinical sensitivity, specificity, or diagnostic accuracy can be drawn at this stage. Second, the accuracy of smartphone-based infrared eye tracking is inherently constrained compared to gold-standard video-oculography (VOG) systems. Dedicated VOG devices provide higher spatial and temporal resolution, controlled illumination, and calibrated optical geometries. In contrast, smartphone-based systems are subject to variable imaging conditions, including sensor noise, motion artifacts, partial occlusions (e.g., eyelids or eyelashes), and changes in ambient illumination. These factors introduce uncertainty in pupil localization, which can be formally expressed in (Equation 5):

$$\hat{p}(t)=p(t)+\epsilon (t),$$ (5)

where ϵ(t) represents measurement noise that propagates into derived velocity and frequency features.

Third, the IMU-based gait analysis relies on simplified biomechanical assumptions and indirect reconstruction of anatomical landmarks. The estimation of position from acceleration signals involves numerical integration, which is known to accumulate represented by (Equation 6):

$$\vec{S}(t)=\int_{0}^{t}\int_{0}^{\tau }a(s)\;ds\;d\tau +C,$$ (6)

where C denotes integration constants. Without external references or zero-velocity updates, this drift can significantly affect the accuracy of reconstructed trajectories and derived metrics such as Triangular Area Variability.

Furthermore, the proposed geometric model assumes a simplified representation of human gait using a limited number of virtual markers (S1, $P_{L}$, $P_{R}$). This abstraction does not account for higher-order biomechanical effects such as joint kinematics, soft tissue artifacts, and inter-segmental dynamics, which are typically captured in marker-based motion capture systems. Another limitation concerns the machine learning component. While the CNN-LSTM architecture is “conceptually suitable” for spatio-temporal pattern recognition, the current work does not include training on large-scale annotated datasets. As a result, the generalization capability of the model remains unverified, and potential issues such as overfitting, domain shift, and class imbalance are not addressed. Finally, practical deployment raises additional challenges, including device heterogeneity, energy consumption, thermal constraints, and data privacy considerations. Real-time processing on mobile hardware requires careful optimization of model complexity and memory usage to ensure stable performance. Future work should therefore focus on (i) clinical validation with patient cohorts, (ii) quantitative benchmarking against established diagnostic modalities such as VOG and optical motion capture (e.g., Vicon System), (iii) sensor fusion approaches to mitigate IMU drift, and (iv) robust machine learning training using diverse, annotated datasets [20]. Addressing these aspects is essential for translating the proposed system from a “conceptual proof-of-concept framework” to a clinically reliable diagnostic tool.

## 7 Conclusion

This work presents a proof-of-concept framework for a smartphone-based system that integrates infrared eye-tracking and IMU-based gait analysis for the computational modeling of vestibular-related signal patterns. By combining computer vision, signal processing, and deep learning techniques within a unified architecture, the system enables the extraction of oculomotor and biomechanical features using widely available consumer hardware. The results illustrate the internal behavior of the proposed approach within a simulation-based setting. Both the eye-tracking and inertial sensing pipelines operate under simulation conditions and produce geometric and signal-based features, such as pupil motion trajectories, frequency-domain characteristics, and stability-related metrics including Triangular Area Variability. The integration of these modalities provides a unified computational representation of multimodal signals within the proposed framework, extending beyond single-sensor analysis at a conceptual level. From a systems perspective, the implementation highlights the potential of modern smartphones as computational platforms for signal processing tasks. On-device processing using GPUs and neural accelerators enables efficient analysis while reducing reliance on external infrastructure [21]. This is relevant in the context of telemedicine, where accessibility and scalability are important considerations. However, the present study is limited to a simulation-based evaluation and does not establish clinical validity or diagnostic performance. The proposed framework should therefore be interpreted as a conceptual foundation for mobile vestibular and gait analysis rather than a validated medical system. Future research will focus on clinical studies involving patient populations, quantitative evaluation against reference measurement systems, and further optimization of both hardware and algorithms. The integration of CNN and LSTM models could additionally support representation learning of spatial and temporal signal dynamics. The learned feature representations $h_{t}$ capture characteristic patterns of simulated eye movement signals, suggesting their potential use for sequence-based modeling tasks within the proposed framework. In summary, the proposed system illustrates the potential of multimodal, smartphone-based sensing for scalable and accessible vestibular signal analysis, providing a basis for future methodological developments in digital health and mobile computation.

## Data Availability

The original contributions presented in the study are included in the article/Supplementary Material, further inquiries can be directed to the corresponding author/s.
